# Upregulation of the inwardly rectifying potassium channel Kir2.1 (KCNJ2) modulates multidrug resistance of small-cell lung cancer under the regulation of miR-7 and the Ras/MAPK pathway

**DOI:** 10.1186/s12943-015-0298-0

**Published:** 2015-03-12

**Authors:** Huanxin Liu, Jie Huang, Juan Peng, Xiaoxia Wu, Yan Zhang, Weiliang Zhu, Linlang Guo

**Affiliations:** Department of Pathology, Zhujiang Hospital, Southern Medical University, Guangzhou, China; Department of Pathology, Guangdong Provincial Corps Hospital of Chinese People’s Armed Police Forces, Guangzhou Medical College, Guangzhou, China; Department of Pathology, the Third Affiliated Hospital of Guangzhou Medical University, Guangzhou, China; Department of Oncology, Zhujiang Hospital, Southern Medical University, Guangzhou, China

**Keywords:** SCLC, KCNJ2/Kir2.1, Chemoresistance, MRP1/ABCC1, miR-7, Ras/MAPK pathway

## Abstract

**Background:**

KCNJ2/Kir2.1, a member of the classical inwardly rectifying potassium channel family, is commonly expressed in a wide range of tissues and cell types. Previous studies indicated that Kir2.1 may be associated with SCLC multidrug resistance (MDR). However, whether Kir2.1 can regulate MDR and its underlying mechanisms remain poorly understood in SCLC.

**Methods:**

KCNJ2/Kir2.1 expression was examined in tissues from fifty-two SCLC cases by immunohistochemistry. Overexpression or knockdown of KCNJ2/Kir21 was performed in multidrug-resistant SCLC cell lines (H69AR and H446AR) and their parental cell lines (H69 and H446) to assess its influence on cell growth, apoptosis, the cell cycle and chemoresistance.

**Results:**

KCNJ2/Kir2.1 was expressed in 44.23% (23/52) of SCLC tissues. Overexpression of KCNJ2/Kir2.1 was correlated with the clinical stage and chemotherapy response in SCLC patients. Knockdown of KCNJ2/Kir2.1 expression using KCNJ2/Kir2.1 shRNA in H69AR and H446AR cells inhibited cell growth and sensitized the cancer cells to chemotherapeutic drugs by increasing cell apoptosis and cell cycle arrest. Forced KCNJ2/Kir2.1 expression in H69 and H446 cells promoted cell growth and enhanced multidrug resistance via reduced drug-induced apoptosis accompanied by cell cycle arrest. KCNJ2/Kir2.1 expression was also influenced by PKC and MEK inhibitors. In addition, multidrug resistance protein 1 (MRP1/ABCC1) was confirmed to interact with KCNJ2/Kir2.1 by Co-IP assays.

**Conclusions:**

KCNJ2/Kir2.1 modulates cell growth and drug resistance by regulating MRP1/ABCC1 expression and is simultaneously regulated by the Ras/MAPK pathway and miR-7. KCNJ2/Kir2.1 may be a prognostic predictor and a potentially novel target for interfering with chemoresistance in SCLC.

**Electronic supplementary material:**

The online version of this article (doi:10.1186/s12943-015-0298-0) contains supplementary material, which is available to authorized users.

## Background

Lung cancer, with 1.35 million new cases and causing more than 1 million deaths each year, is the most common cancer and the leading cause of cancer-related deaths worldwide [1]. Small-cell lung cancer (SCLC), the most aggressive type of lung cancer, constitutes approximately 15-18% of all lung cancers [2]. According to the Veterans Administration Lung Group system, SCLC is traditionally defined by a two-stage classification system that includes limited disease and extensive disease. At present, chemotherapy remains the first treatment option for SCLC patients. Although 80-90% of SCLC patients are initially responsive to chemotherapy, most of them succumb to the disease within a year due to rapidly developing multidrug resistance (MDR) [3,4]. Thus, MDR has become the main obstacle to the treatment of SCLC and a central issue in improving its prognosis.

Kir2.1, encoded by the *KCNJ2* gene, is a member of the classical inwardly rectifying potassium channel family (Kir2 subfamily). It conducts a strong inward rectifier K^+^ current in a wide range of tissues and cell types, including neurons, skeletal muscle, cardiac myocytes, and immune system and carcinoma cells [5]. The *KCNJ2* gene was first cloned by Kubo et al. from a macrophage cell line in 1993 [6]. Similar to the other members of the Kir family, Kir2.1 is tetrameric, containing two transmembrane helix domains (M1 and M2), an ion-selective P-loop between M1 and M2, and cytoplasmic N- and C-terminal domains. Functionally, Kir2.1 plays a key role in maintaining the resting membrane potential and regulating cellular excitability in SCLC cells, cardiac myocytes, skeletal muscle and neurons [7-9]. Changes in the expression levels of K^+^ channels induced by aberrant *KCNJ2* expression have substantial effects on cellular processes such as cell death, apoptosis, proliferation and adhesion, which is linked to a variety of cardiac and neurological disorders [10-15]. Human SCLC cells are suggested to be of neurorctodermal origin and exhibit electrophysiological characteristics typical of neuroendocrine cells. Previous studies have indicated that the large, inwardly rectifying K+ current is generated by Kir2.1 and may be associated with SCLC cell MDR [16,17]. However, whether Kir2.1 can regulate MDR and its underlying mechanisms remain poorly understood in SCLC.

MicroRNAs (miRNAs) are a class of small, non-coding RNAs of 18–24 nucleotides in length that negatively regulate the expression of specific genes by binding to the 3′ untranslated region (3’UTR) of an mRNA, leading to either translational inhibition or mRNA degradation [18]. Recent evidence has shown that more than 50% of miRNAs are located in cancer-associated genomic break points and can function as tumor suppressor genes or oncogenes depending on their targets [19,20]. Moreover, extensive studies have indicated that miRNAs are closely related to responses to chemotherapeutic treatment [21-24]. For example, Yang et al. reported that miR-214 induced cell survival and cisplatin resistance in ovarian cancer [25]. Additionally, miR-650 levels affected the chemosensitivity of lung adenocarcinoma cells to docetaxel via Bcl-2/Bax expression regulation by directly targeting ING4 [23], and suppression of miR-137 expression in a drug-resistant SCLC cell line increased its sensitivity to cisplatin [26]. Moreover, our previous miRNA expression profile study revealed that the expression of 61/852 miRNAs was significantly increased (>3-fold) in MDR SCLC H69AR cells compared with their drug-sensitive parental cell line H69, suggesting a role for these differentially expressed miRNAs in the development of drug resistance in SCLC cells [22].

We previously found that KCNJ2 is overexpressed in H69AR cells compared to parental H69 cells, whereas miR-7 is expressed at a lower level in H69AR cells compared with H69 cells (unpublished data). In the present study, we further investigated the roles of KCNJ2/Kir2.1 in drug resistance using human drug-resistant SCLC cell lines (H69AR and H446AR). The correlation between KCNJ2 expression and clinical drug response was analyzed in SCLC patients. We then validated the interaction between Kir2.1 and MRP1/ABCC1 by co-immunoprecipitation (Co-IP). Furthermore, we showed that KCNJ2 was modulated by the Ras/MAPK pathway and directly targeted by miR-7. Collectively, our results provide a novel explanation for the chemoresistance of SCLC and suggest that KCNJ2/Kir2.1 plays a crucial role in SCLC MDR.

## Results

### Kir2.1 expression is associated with the clinical stage and the chemotherapy Response of SCLC patients

**Figure 1:**
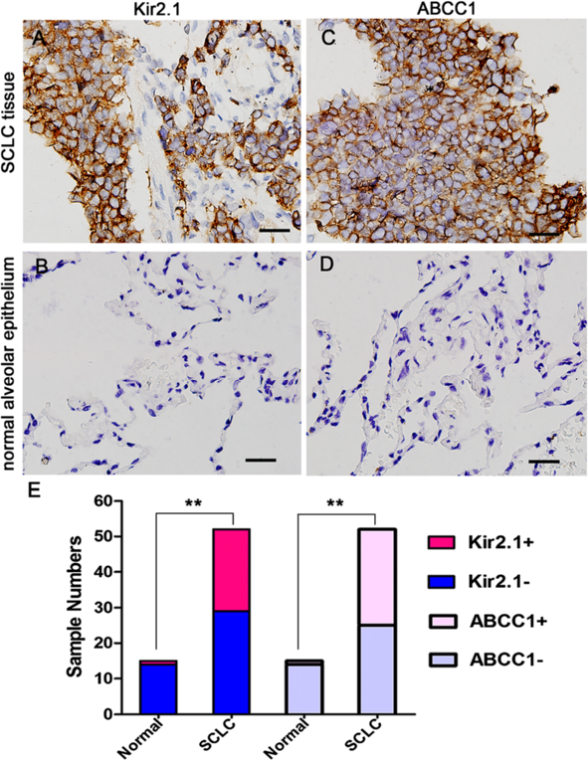
**Kir2.1 and MRP1/ABCC1 expression in SCLC and normal alveolar epithelium tissues by IHC. (A-B)** Representative images of Kir2.1 IHC staining in SCLC **(A)** and normal alveolar epithelium tissues **(B)**. **(C-D)** Representative images of MRP1/ABCC1 IHC staining in SCLC **(C)** and normal alveolar epithelium tissues **(D)**. Brown chromogen represents positive staining. Scale bar, 50 μm. **(E)** The IHC staining results of Kir2.1 and MRP1/ABCC1 expression in normal alveolar epithelium and SCLC tissues. The positive rate of Kir2.1 and MRP1/ABCC1 were higher in SCLC tissues than normal alveolar epithelium. **, *P* < 0.01 compared with normal alveolar epithelium.

To investigate the clinical features of Kir2.1 expression in SCLC, we first examined the expression levels of Kir2.1 in 52 SCLC specimens and 15 normal lung tissues by immunohistochemistry (IHC). Kir2.1 was localized on the membrane of the cancer cells (Figure 1A), whereas no positive Kir2.1 staining was observed in normal lung alveolar epithelium (Figure 1B). And the corresponding IHC staining scores are indicated in Additional file 1A: Figure S1A. Additionally, the positive expression of Kir2.1 was 23 of 52 (44%) in SCLC and 1 of 15 in normal bronchial epithelium, respectively (Figure 1E).

We further analyzed the correlation between Kir2.1 expression and the clinicopathological features of SCLC patients. As shown in Table 1, Kir2.1 expression was significantly more frequent at the extensive disease stage and in the drug-resistant group than at the limited disease stage and in the drug-sensitive group. Correlation analysis showed that Kir2.1 expression was significantly associated with clinical stage and chemotherapy response in SCLC patients (*P* < 0.05), but not with gender, age or smoking history (*P* > 0.05) (Table 1).

**Table 1:** **The expression of Kir2.1 and MRP1/ABCC1 and their relationships with the clinicopathological characteristics in SCLC patients**

**Clinic pathological features**	**n**	**Expression of Kir2.1**			**Expression of MRP1/ABCC1**		
		**+**	**-**	***P***	**+**	**-**	***P***
**Gender**				1			1
Male	43	18	25		21	22	
Female	9	5	4		6	3	
**Age**				0.892			0.426
<55^†^	23	7	16		10	13	
≥55	29	16	13		17	12	
**Smoking history**				1			0.328
Yes	41	20	21		22	19	
No	11	3	8		5	6	
**Clinical stage**				0.032			0.001
limited disease	25	7	18		6	19	
extensive disease	27	16	11		21	6	
**Chemotherapy reponse**				0.021			0.001
Sensitive group	25	6	19		7	18	
Resistant group	22	17	5		20	2	

^†^median age.

-, negative expression; +, positive expression.

The P-value was calculated by *χ* ^2^ test.

### KCNJ2/Kir2.1 expression is correlated with chemoresistance in SCLC

**Figure 2:**
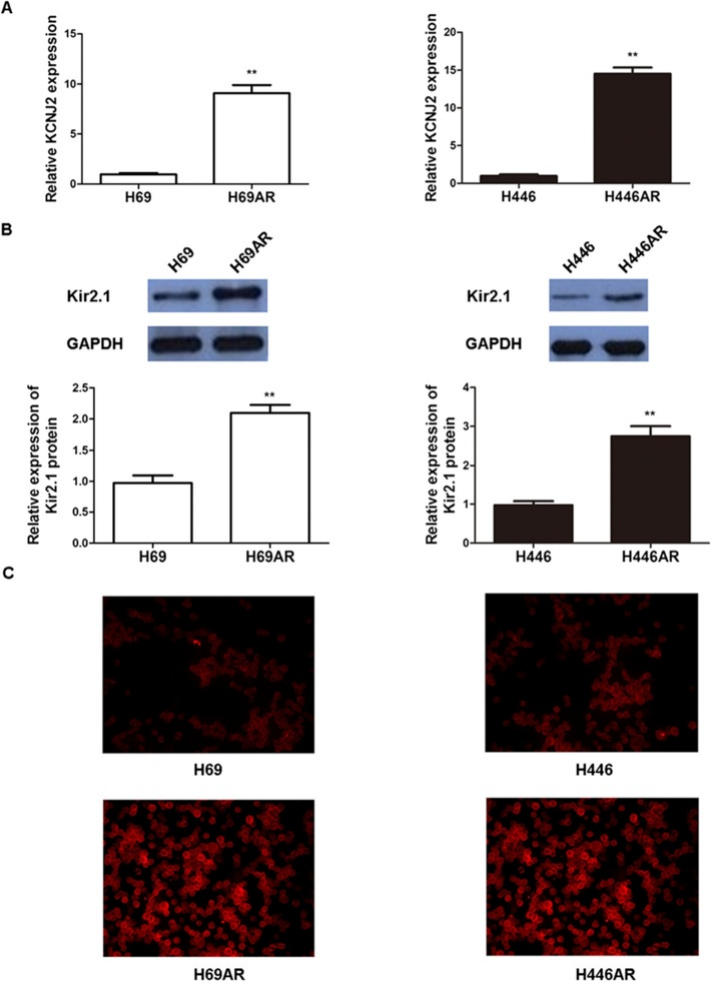
**KCNJ2/Kir2.1 expressionin SCLC cells. (A)** KCNJ2 mRNA level was determined by qRT-PCR in SCLC multi-drug resistant H69AR and H446AR cells and their corresponding parental cells, H69 and H446. **(B-C)** Western blotting and immunofluorescence analyses showed that Kir2.1expression was upregulatedin H69ARand H44AR cellsas compared with H69 and H446 cells, respectively. Values of three independent experiments are represented as the mean ± SD. **, *P* < 0.01 compared with the parental cells.

Our previous study indicated a 4.75-fold upregulation of KCNJ2 expression in H69AR cells compared with their parental H69 cells by cDNA microarray analysis (unpublished data). We confirmed the result in two pairs of SCLC cells (H69AR/H69 and H446AR/H446) by qRT-PCR, western blotting and immunofluorescence assays. Figure 2 shows that KCNJ2/Kir2.1 expression was significantly increased at both mRNA and protein levels in H69AR and H446AR cells as compared with H69 and H446 cells, respectively.

**Figure 3:**
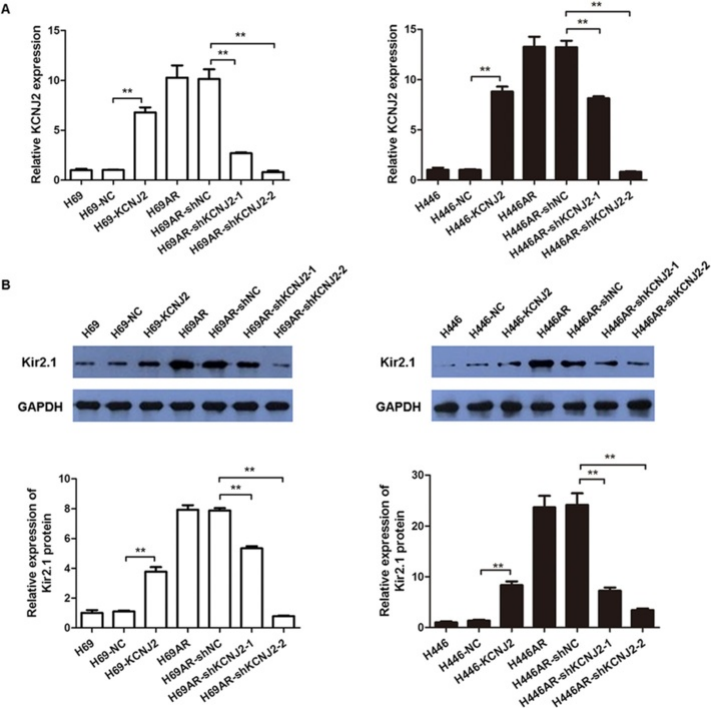
**Altered KCNJ2/Kir2.1 expression in SCLC cells.** qRT-PCR **(A)** and Western blot **(B)** analyses showed forced KCNJ2/Kir2.1 expression in H69 and H446 cells after transfection of pcDNA3.1-KCNJ2, and KCNJ2/Kir2.1 inhibition in H69AR and H446AR cells by shKCNJ2-1or shKCNJ2-2. The bars represent the means ± SD of three independent experiments. **, *P* < 0.01 compared withthe corresponding NC cells transfected with pcDNA3.1 empty vector or shNC.

Based on the upregulation of KCNJ2/Kir2.1 in MDR SCLC cells, we hypothesized that KCNJ2/Kir2.1 may be involved in SCLC chemoresistance. To test this hypothesis, we performed gain-of-function and loss-of-function studies. First, we developed two stable KCNJ2/Kir2.1-overexpressing sublines, H69-KCNJ2 and H446-KCNJ2, by transfecting H69 and H446 cells with pcDNA3.1-KCNJ2, and their corresponding negative control sublines (H46-NC and H446-NC) that were transfected with pcDNA3.1 empty vector. qRT-PCR and western blotting analyses showed that KCNJ2/Kir2.1 expression was significantly upregulated at both the mRNA and protein levels in H69-KCNJ2 and H446-KCNJ2 as compared with H46-NC and H446-NC, respectively (Figure 3). Subsequently, we stably transfected multidrug-resistant H69AR and H446AR cells with two shRNAs (shKCNJ2-1 and shKCNJ2-2) targeting KCNJ2 to inhibit KCNJ2/Kir2.1 expression. As shown in Figure 3, KCNJ2/Kir2.1 expression was notably decreased in cells transfected with shKCNJ2-1 or shKCNJ2-2 (H69AR-shKCNJ2-1, H69AR-shKCNJ2-2, H446AR-shKCNJ2-1 and H446AR-shKCNJ2-2) when compared with their corresponding negative controls, H69AR-shNC or H446AR-shNC. The viability and sensitivity of the SCLC cells to chemotherapeutic drugs (ADM, CDDP and VP-16) were then assessed using CCK8 assays. Table 2 shows that the IC_50_ values of H69 and H446 cells significantly increased after transfection with pcDNA3.1-KCNJ2, whereas the IC_50_ values of H69AR and H446AR cells decreased after the introduction of shKCNJ2-1 or shKCNJ2-2. Collectively, these results indicated that KCNJ2/Kir2.1upregulation or downregulation could significantly affect the sensitivity of SCLC cells to chemotherapeutic drugs, suggesting that KCNJ2/Kir2.1 expression may be associated with chemoresistance in SCLC.

**Table 2:** **Effect of KCNJ2/Kir2.1 on drug sensitivity** $\left(\overline{\mathbf{\mathsf{X}}}\pm \kern0.5em \mathbf{\mathsf{s}},\kern0.5em \mathbf{n}=\mathbf{\mathsf{3}},\kern0.5em \mathbf{\mathsf{\boldsymbol{\upmu}g}}/\mathbf{\mathsf{ml}}\right)$

**Cells**	**IC50**		
	**ADM**	**CDDP**	**VP-16**
H69	5.222 ± 0.117	17.711 ± 0.279	124.03 ± 2.791
H69-NC	5.426 ± 0.87	17.548 ± 0.423	122.27 ± 2.938
H69-KCNJ2	13.678 ± 0.195*	22.649 ± 7.623*	283.41 ± 6.884*
H446	4.293 ± 0.049	4.529 ± 0.063	52.342 ± 3.0
H446-NC	4.412 ± 0.212	4.884 ± 0.068	50.296 ± 1.53
H446-KCNJ2	14.048 ± 0.242*	15.303 ± 0.712*	80.064 ± 4.97*
H69AR	211.06 ± 6.665	76.652 ± 1.238	531.58 ± 6.06
H69AR-shNC	209.69 ± 3.157	77.32 ± 1.362	529.72 ± 4.704
H69AR-shKCNJ2-1	62.275 ± 1.214*	44.583 ± 1.514*	394.74 ± 19.996*
H69AR-shKCNJ2-2	18.823 ± 0.935*	33.183 ± 2.637*	359.51 ± 8.62*
H446AR	39.29 ± 0.738	133.73 ± 4.875	179.63 ± 2.978
H446AR-shNC	42.35 ± 1.388	132.12 ± 4.434	175.64 ± 8.726
H446AR-shKCNJ2-1	27.082 ± 2.289*	72.724 ± 0.896*	118.31 ± 4.934*
H446AR-shKCNJ2-2	19.920 ± 0.688*	21.517 ± 2.693*	108.51 ± 5.772*

*P < 0.05 (compared with corresponding negative control groups).

### KCNJ2/Kir2.1 induces cell cycle arrest and apoptosis following exposure to chemotherapeutic drugs

The above observations prompted us to investigate the possible mechanisms of KCNJ2/Kir2.1 in SCLC chemoresistance. Using flow cytometry analysis, we evaluated the effect of KCNJ2/Kir2.1 on cell cycle control and apoptosis following exposure to chemotherapeutic drugs.

**Figure 4:**
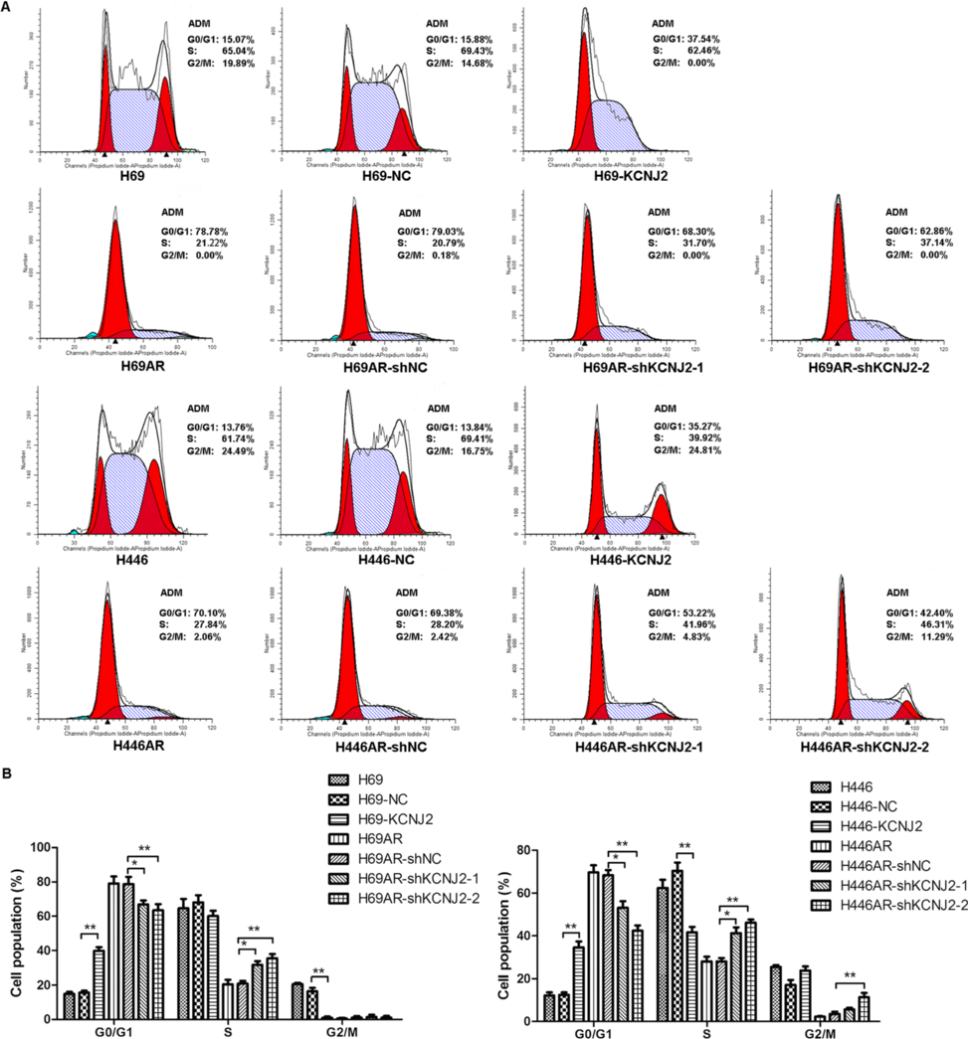
**KCNJ2/Kir2.1 affects the cell cycle distribution of SCLC cells after ADM treatment. (A)** Representative FACS profiles are shown. The numbers indicate the percentages of cells in the G0/G1, S and G2/M phases. **(B)** Histographs for the cell cycle distribution of SCLC cells. H69 and H446 cells stably transfected with KCNJ2 expression plasmid (H69-KCNJ2 and H446-KCNJ2) showed an increased proportion in the G0/G1 phase and a decreased proportion in the S phase when compared with H69-NC and H446-NC, respectively. KCNJ2/Kir2.1-knockdown cells (H69AR-shKCNJ2-1, H69AR-shKCNJ2-2, H446AR-shKCNJ2-1 and H446AR-shKCNJ2-2) showed a decreased proportion in the G0/G1 phase and an increased proportion in the S phase when compared with H69AR-shNC or H446AR-shNC. Data are shown as the means ± SD from three independent experiments. *, *P* < 0.05; **, *P* < 0.01 compared with the corresponding NC cells transfected with pcDNA3.1 empty vector or shNC.

As shown in Figure 4, overexpression of KCNJ2/Kir2.1 in H69 and H446 cells induced an increase of cells entering the G0/G1 phase and a corresponding decline of cells in the S phase after treatment with ADM (4.6 μM) for 24 h. In contrast, knockdown of KCNJ2/Kir2.1 by shKCNJ2-1 or shKCNJ2-2 in H69AR and H446AR cells resulted in a significant decrease of cells in the G0/G1 phase and an increase of cells in the S phase. These results suggest that KCNJ2/Kir2.1could induce cell cycle arrest.

**Figure 5:**
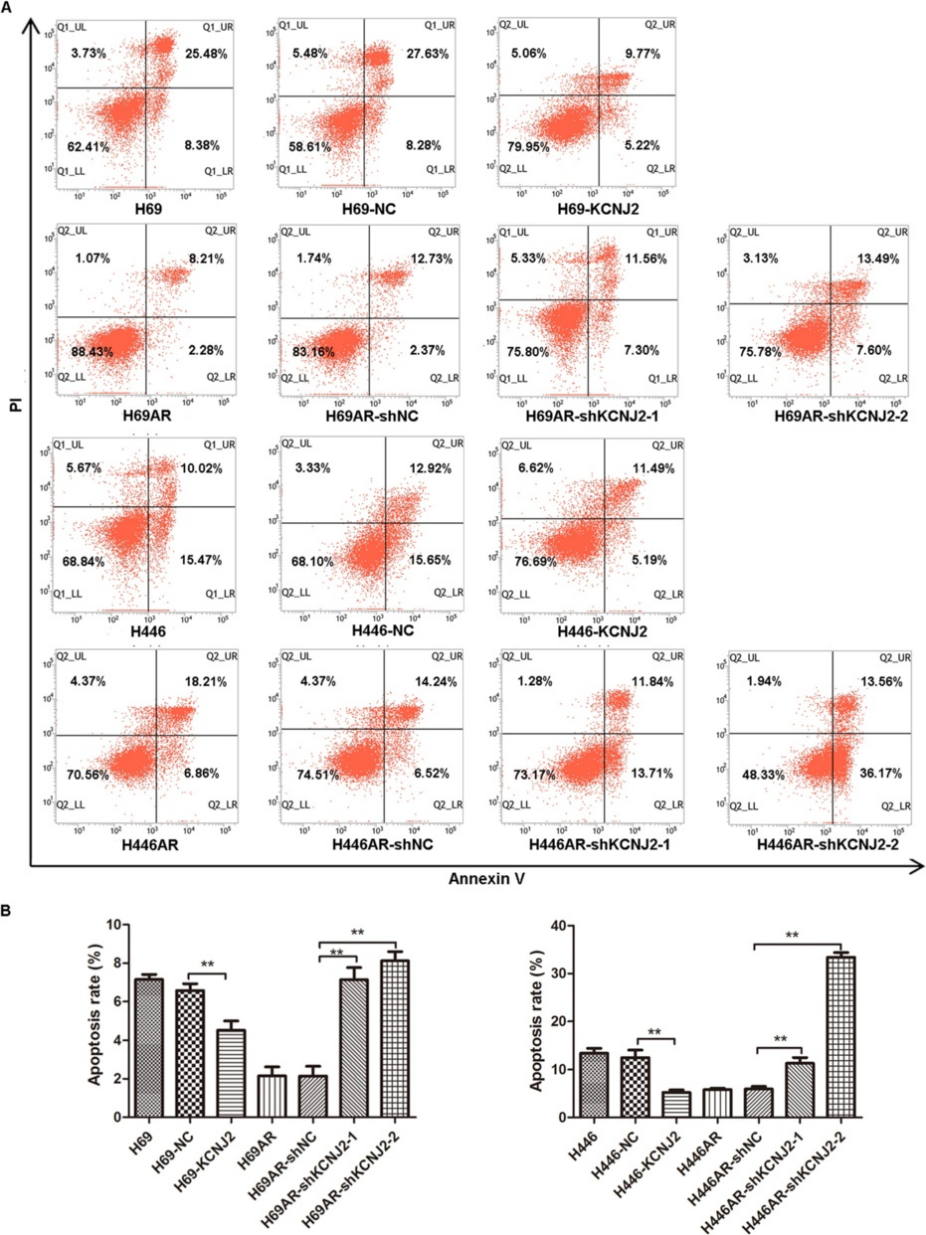
**KCNJ2/Kir2.1 affects cell apoptosis in SCLC cells after ADM treatment. (A)** Cells were analyzed by annexin-V/propidium iodide (PI) dual labeling. Representative FACS profiles are shown, on which cell population in the quadrant of Annexin V^+^PI^−^represents apoptotic cells. **(B)** Histographs for cell apoptosis of SCLC cells. The percentage of the Annexin V^+^PI^−^cell population was determined. The results show data from at least three independent experiments. **, *P* < 0.01 compared with the corresponding NC cells.

We further examined the impact of KCNJ2/Kir2.1 on apoptosis. Notably, after treatment with ADM (4.6 μM) for 24 h, upregulating KCNJ2/Kir2.1 expression in H69 and H446 cells prevented drug-induced apoptosis. Meanwhile, silencing KCNJ2/Kir2.1 expression in H69AR and H446AR cells significantly increased drug-induced apoptosis (Figure 5). In addition, similar results of cell cycle and apoptosis analysis were observed in SCLC cells after treatment with CDDP (16.7 μM) or VP-16 (34 μM) (Additional file 2: Figure S2, Additional file 3: Figure S3, Additional file 4: Figure S4, and Additional file 5: Figure S5).

### KCNJ2/Kir2.1 enhances SCLC tumor growth *in vivo*

**Figure 6:**
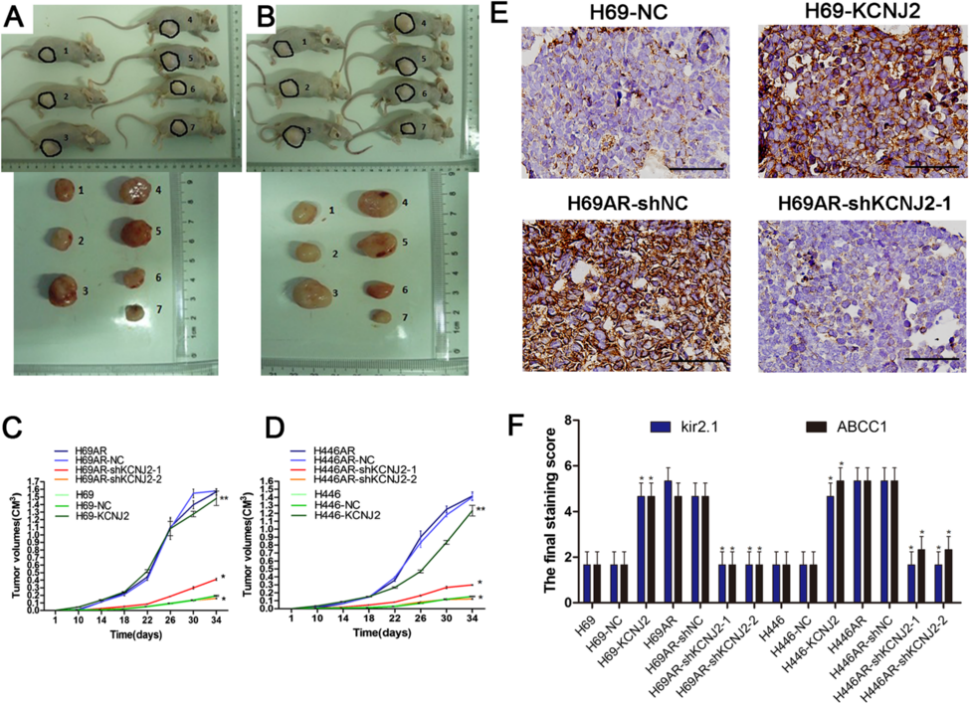
**KCNJ2/Kir2.1 enhances SCLC tumor growth** ***in vivo*** **. (A, C)** Tumors formation and growth curves of tumor volumes formed by H69 and H69AR cells with altered KCNJ2 expression.1, H69; 2, H69-NC; 3, H69-KCNJ2; 4, H69AR; 5, H69AR-NC; 6, H69AR-shKCNJ2-1; 7, H69AR-shKCNJ2-2. **(B, D)** Tumors formation and growth curves of tumor volumes formed by H446 and H446AR cells with altered KCNJ2 expression. 1, H446; 2, H446-NC; 3, H446-KCNJ2; 4, H446AR; 5, H446AR-NC; 6, H446AR-shKCNJ2-1; 7, H446AR-shKCNJ2-2. **(E)** Kir2.1 expression was examined by IHC in the tumor xenografts originating from H69-NC, H69-KCNJ2, H69AR-NC and H69AR-shKCNJ2-1 cells. Representative images were shown. Brown chromogen represents positive staining. Scale bar, 50 μm. **(F)** Kir2.1 and MRP1/ABCC1expression were evaluated by staining scores in the tumor xenografts. *, *P* < 0.05 compared with the corresponding NC cells transfected with pcDNA3.1 empty vector or shNC.

To further determine the effect of KCNJ2/Kir2.1 on tumor growth *in vivo*, SCLC cells with altered KCNJ2/Kir2.1 expression were subcutaneously injected into nude mice. As shown in Figure 6A-D, the injection of KCNJ2/Kir2.1-overexpressing H69 and H446 cells resulted in significantly increased tumor weights compared with the injection of the corresponding NC cells. However, with KCNJ2/Kir2.1-knockdown H69AR and H446AR cells, the average tumor weight decreased to 12-25% of that of the tumors originating from the cells transfected with shNC. Additionally, the injection of KCNJ2/Kir2.1-overexpressing H69 and H446 cells resulted in earlier tumor formation compared with the injection of NC cells. In contrast, the time until tumor appearance was prolonged in H69AR and H446AR cells transfected with shKCNJ2-1 or shKCNJ2-2 compared with that transfected with shNC. These findings demonstrated that KCNJ2/Kir2.1 enhanced SCLC tumor growth *in vivo*.

We then further examined Kir2.1 expression in SCLC tissues from the tumor xenografts by IHC. The results showed higher Kir2.1 expression in H69-KCNJ2 tumors and lower expression in shKCNJ2-1 H69AR tumors as compared with the corresponding NC cells (Figure 6E and Additional file 1B: Figure S1B). Moreover, evaluated by staining scores, Kir2.1 and MRP1/ABCC1 expression were significantly upregulated in tumors originating from H69 and H446 cells transfected with KCNJ2-overexpressing vector and downregulated in tumors of H69AR and H446AR cells transfected with shKCNJ2-1 or shKCNJ2-2 (Figure 6F).

### KCNJ2/Kir2.1 interacts with MRP1/ABCC1

We previously found that the expression of both KCNJ2/Kir2.1 and MRP1/ABCC1 was increased in H69AR cells compared with parental H69 cells by cDNA microarray analysis [22]. To further investigate the relationship between KCNJ2/Kir2.1 and MRP1/ABCC1 in SCLC, we simultaneously detected the expression of these two proteins in SCLC and normal lung tissues by IHC. Membrane localization of KCNJ2/Kir2.1 and MRP1/ABCC1 was observed in cancer cells (Figure 1A and C), whereas no positive MRP1/ABCC1 staining was observed in normal lung alveolar epithelium (Figure 1D). Similar to Kir2.1, the rate of MRP1/ABCC1 positivity was much higher in SCLC specimens (52%) than in normal bronchial epithelium (7%) (Figure 1E). Correlation analysis showed that MRP1/ABCC1 expression was significantly associated with the SCLC patient’s clinical stage and chemotherapy response, but not with gender, smoking, age or lymph node metastasis (Table 1). Furthermore, as shown in Table 3, MRP1/ABCC1 expression was clearly correlated with Kir2.1 expression in SCLC tissues.

**Table 3:** **The correlation between Kir2.1 and MRP/ABCC1 in SCLC tissues**

**ABCC1**	**Kir2.1**		***χ*** ^**2**^	***P***
	**-**	**+**		
**-**	23	2	25.622	<0.001
**+**	6	21		

-, negative expression; +, positive expression.

The P-value was calculated by *χ* ^2^ test.

**Figure 7:**
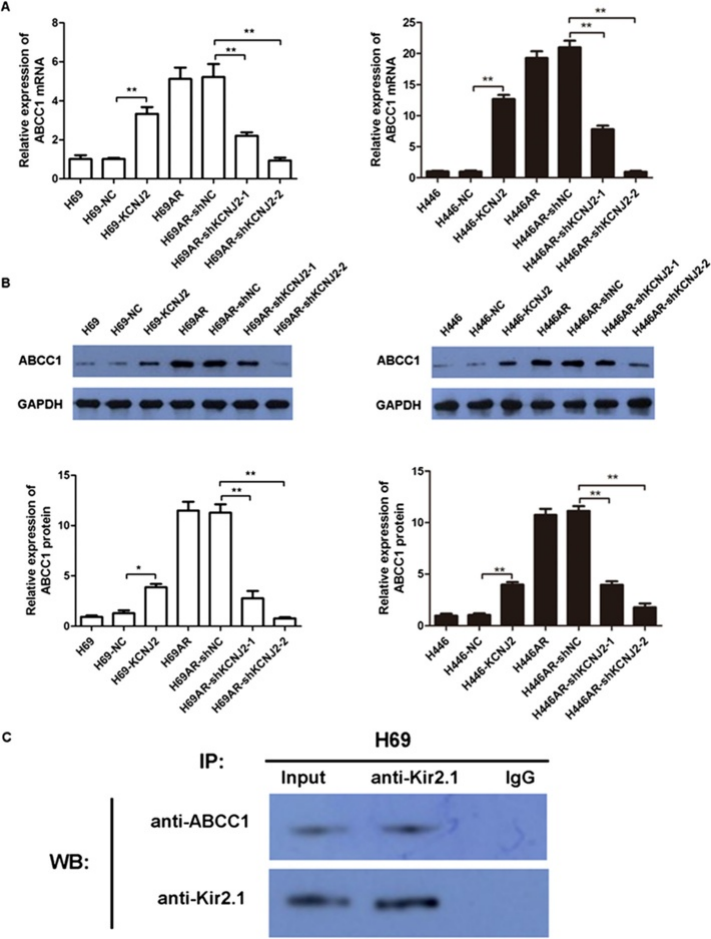
**Kir2.1 interacts with MRP1/ABCC1 expression. (A-B)** qRT-PCR **(A)** and Western blotting **(B)** analyses showed that KCNJ2/Kir2.1 upregulation or downregulation successfully elevated or suppressed MRP1/ABCC1 mRNA and protein levels, respectively. **(C)** The interaction between Kir2.1 and MRP1/ABCC1 was analyzed by Co-IP. Anti-Kir2.1 antibody was used for IP. The amounts of ABCC1 and Kir2.1 in the immunoprecipitates were detected by Western blotting with the indicated specific antibodies.

To explore the relationship between KCNJ2/Kir2.1 and MRP1/ABCC1, we analyzed MRP1/ABCC1 expression changes with upregulated or downregulated KCNJ2/Kir2.1 expression. qRT-PCR and western blotting analyses revealed a marked increase in MRP1/ABCC1 in H69 and H446 cells transfected with pcDNA3.1-KCNJ2 and a decrease in MRP1/ABCC1in H69AR and H446AR cells transfected with shKCNJ2-1 or shKCNJ2-2 (Figure 7A-B). These results provide evidence indicating that KCNJ2/Kir2.1 could affect MRP1/ABCC1 expression.

We performed Co-IP assays to further evaluate the interaction between KCNJ2/Kir2.1 and MRP1/ABCC1 in SCLC cells. Cell lysates from H69 cells were prepared and immunoprecipitated with an anti-Kir2.1 antibody. Both immunoprecipitates and cell lysates without IP were blotted with anti-ABCC1 and anti-Kir2.1. As shown in Figure 7C, Kir2.1 formed a complex with ABCC1. These data reveal that Kir2.1 can interact with MRP1/ABCC1.

### The modulatory effect of the Ras/MAPK pathway on KCNJ2/Kir2.1

**Figure 8:**
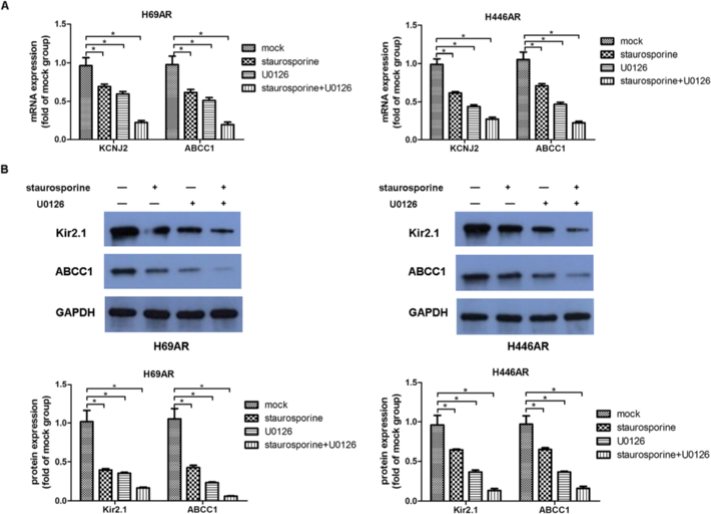
**The Ras/MAPK pathway modulates the expression of KCNJ2/Kir2.1. (A-B)** qRT-PCR and Western blot analyses showed that KCNJ2/Kir2.1 expression was inhibited at the mRNA and protein levels in H69AR and H446AR cells treated with the PKC inhibitor staurosporine, the MEK inhibitor U0126, or staurosporine plus U0126. *, *P* < 0.05 compared with the corresponding mock cells.

Based on previous findings that the Ras-MAPK pathway is involved in modulating the inward rectifier potassium channel IRK1 [5], we investigated whether the Ras-MAPK pathway could regulate KCNJ2/Kir2.1 in SCLC. Initially, SCLC MDR H69AR and H446AR cells were treated with PKC inhibitor (staurosporine, 10 nM) and MEK inhibitor (U0126, 10 μM) to inhibit two key components of Ras-MAPK pathway respectively. Then, we examined the mRNA and protein levels of both KCNJ2/Kir2.1 and MRP1/ABCC1 in these SCLC cells by qRT-PCR and Western blotting. The results showed that the expression of both KCNJ2/Kir2.1 and MRP1/ABCC1 was significantly downregulated following treatment with staurosporine and U0126 as compared with the controls (Figure 8). Moreover, an additive inhibitory effect on KCNJ2/Kir2.1 and MRP1/ABCC1 expression was observed in SCLC cells upon simultaneous PCK and MEK suppression (Figure 8). These findings suggest that KCNJ2/Kir2.1 modulation is mediated by the Ras-PKC-MAPK pathway.

### MiR-7 directly regulates KCNJ2 expression in SCLC

**Figure 9:**
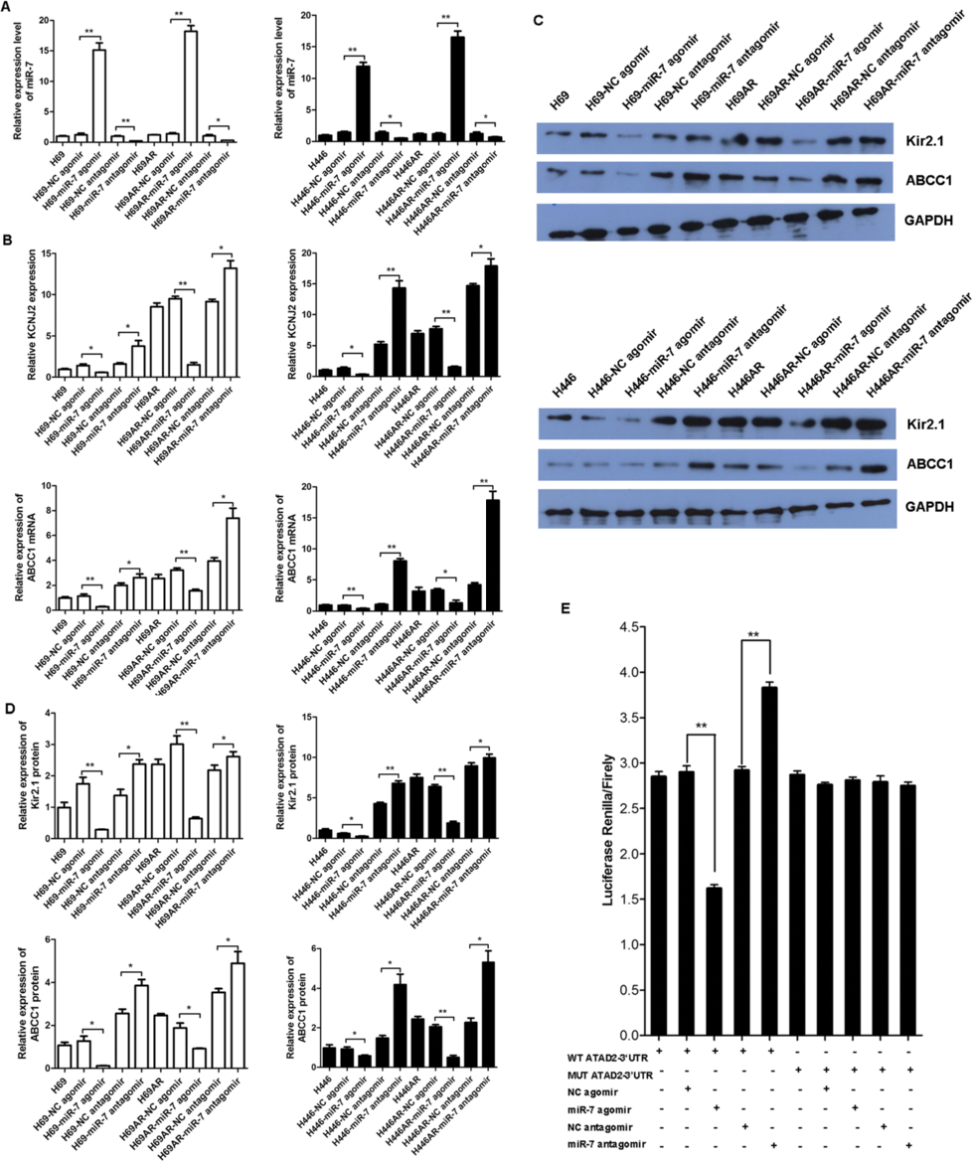
**KCNJ2 is a direct target of miR-7. (A)** The miR-7levels in H69, H69AR, H446, H446AR cells were measured after transfection with an miR-7 agomir or antagomir or the corresponding NC. **(B-D)** KCNJ2/Kir2.1 and MRP1/ABCC1expression at mRNA and protein levels was assessed after transfection of SCLC cells with an miR-7 agomir or antagomir or the corresponding NC. **(E)** A dual luciferase assay was performed in H69 cells transfected with the luciferase construct alone or cotransfected with anmiR-7 agomir or antagomir or the corresponding NC. *, *P* < 0.05; **, *P* < 0.01 compared with the corresponding NC cells transfected with NC agomir or NC antagomir.

All processes involved in cancer, including apoptosis and proliferation, have been shown to be regulated by small regulatory non-coding RNAs, i.e., miRNAs [27]. Therefore, the above results showing that KCNJ2/Kir2.1 is overexpressed in SCLC led us to hypothesize that KCNJ2/Kir2.1 expression may be regulated by endogenous miRNAs. We utilized three prediction algorithms (PicTar, TarScan and miRBase database) to predict the miRNAs that possibly target KCNJ2 in SCLC. The results showed that miR-7, miR-212 and miR-1 have potential interaction sites in the 3’-untranslated region (3’UTR) of KCNJ2 mRNA. Based on the involvement of miR-7 in drug resistance, we investigated whether KCNJ2 is regulated by miR-7. We transfected an miR-7 agomir or antagomir into SCLC cells to increase or decrease miR-7 expression, respectively (Figure 9A). The levels of KCNJ2/Kir2.1 and MRP1/ABCC1 were then measured by qRT-PCR and Western blotting at 48 h post-transfection. The expression of both KCNJ2/Kir2.1 and MRP1/ABCC1 decreased significantly after transfection of miR-7 agomir compared with NC agomir, whereas the miR-7 antagomir clearly upregulated the levels of KCNJ2/Kir2.1 and MRP1/ABCC1 when compared with NC antagomir (Figure 9B-D).

To test whether miR-7 can regulate KCNJ2/Kir2.1 via direct interaction with the KCNJ2 3’UTR, we constructed luciferase reporter vectors containing wildtype (psiCHECK2-KCNJ2-wt) or mutated (psiCHECK2-KCNJ2-mt) 3’UTRs of KCNJ2mRNA. H69 cells were cotransfected with each vector and either the miR-7 agomir or antagomir or the corresponding NC; the KCNJ2 luciferase activity was measured after cotransfection. As shown in Figure 9E, the miR-7 agomir suppressed luciferase activity when cotransfected with the wildtype reporter vector as compared with NC agomir, whereas the miR-7 antagomir increased luciferase activity compared with NC antagomir. In contrast, the luciferase activity of the mutated reporter vector was not affected by simultaneous transfection with the miR-7 agomir or antagomir (Figure 9E). Taken together, these findings suggest that KCNJ2 is a direct target of miR-7 in SCLC.

### Regulation of chemoresistance by KCNJ2/Kir2.1 is partly mediated by miR-7

**Figure 10:**
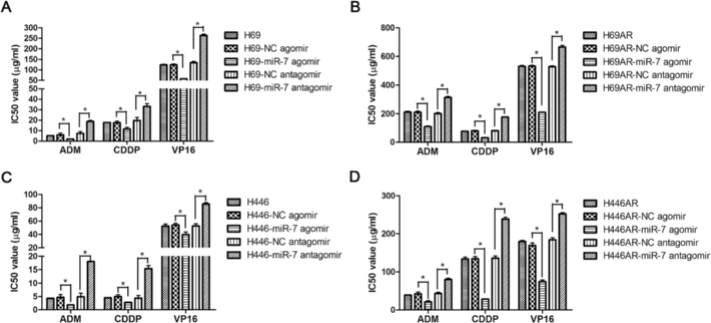
**The effect of miR-7 on drug sensitivity. (A-B)** The sensitivity of H69 and H69AR cells to chemotherapeutic drugs (ADM, CDDP and VP-16) after transfected with the miR-7 agomir, antagomir or the corresponding NC. **(C-D)** The sensitivity of H446 and H446AR cells to chemotherapeutic drugs (ADM, CDDP and VP-16) after transfected with the miR-7 agomir, antagomir or the corresponding NC. ADM, Adriamycin; CDDP, Cisplatin; VP-16, Etoposide. *P < 0.05 (compared with corresponding negative control groups).

To further investigate the effect of miR-7 on the chemoresistance of SCLC cells, we analyzed the sensitivity of SCLC cells to chemotherapeutic drugs (ADM, CDDP and VP-16) after transfection with the miR-7 agomir, antagomir or the corresponding NC. The IC_50_ values of the chemotherapeutic drugs were significantly decreased after miR-7 agomir transfection compared with the controls. Meanwhile, the IC_50_ values increased when expression of miR-7 was suppressed by miR-7 antagomir (Figure 10). These results demonstrated that the upregulation of miR-7 expression sensitized the SCLC cells to all three chemotherapeutic drugs, whereas the downregulation of miR-7 desensitized the SCLC cells. Together, these data suggest that miR-7 deregulation may be responsible for the effects of KCNJ2 on SCLC chemoresistance.

### miR-7 expression is associated with Kir2.1 levels and prognosis in SCLC patients

To further confirm the association between miR-7 and KCNJ2/Kir2.1 expression, we analyzed miR-7 expression in the above-mentioned 52 SCLC tissue specimens by qRT-PCR. The median value of miR-7 expression was chosen as the cut-off point for separating SCLC tissues with low-level miR-7 expression (n = 26) from those with high-level miR-7 expression (n = 26). Correlation analysis showed that miR-7 expression was inversely correlated with KCNJ2/kir2.1 (*P* < 0.001) and MRP1/ABCC1 expression (*P* = 0.002) (Table 4). Moreover, low-level miR-7 expression was significantly associated with a more aggressive clinical stage (*P* = 0.012) (Table 4). Kaplan-Meier analysis revealed that SCLC patients with low miR-7 expression had much shorter overall survival times than patients with high miR-7 expression (Additional file 6: Figure S6). In addition, miR-7 expression reached significance in a univariate Cox proportional hazards regression model (*P* = 0.024, Additional file 7: Table S1).

**Table 4:** **Relationship between miR-7 expression and clinicopathological characteristics in SCLC patients**

**Characteristics**	**Total**	**MiR-7 expression**		***χ*** ^**2**^	***P*** **value**
		**-**	**+**		
**Gender**				0.134	0.714
Male	43	22	21		
Female	9	4	5		
**Age**				0.115	0.734
<55^†^	11	6	5		
≥55	41	20	21		
**Stage**				6.240	0.012
Limited disease	25	8	17		
Extensive disease	27	18	9		
**Drug-sensitivity**				3.733	0.053
Sensitive group	25	10	15		
Resistant group	22	15	7		
**Kir2.1**				13.175	<0.001
Negative	29	8	21		
Positive	23	18	5		
**ABCC1**				9.321	0.002
Negative	25	7	18		
Positive	27	19	8		

^†^median age.

-, low expression; +, high expression.

The P-value was calculated by *χ* ^2^ test.

## Discussion

Previous studies have revealed that K+ channel blockers inhibit SCLC cell proliferation via membrane depolarization [16,28] and that some types of inwardly rectifying K^+^channels are involved in SCLC MDR [16,29]. In our previous analysis of a cDNA microarray [22], KCNJ2 expression was increased in SCLC multi-drug resistant H69AR cells compared with the parent H69 cell line, suggesting that KCNJ2/Kir2.1 might be relevant in the drug resistance of SCLC. However, the molecular mechanism by which KCNJ2/Kir2.1 exerts a role in the chemoresistance of SCLC was not clear until now. In this study, we showed that the mRNA and protein expression of KCNJ2/Kir2.1 was upregulated in both H69AR and H446AR cells compared with that in their respective parental cells, confirming the results of the SCLC mRNA expression profiling. Moreover, Kir2.1 expression in 52 clinical SCLC tissues was significantly associated with the chemotherapeutic responses of the SCLC patients. To further investigate whether KCNJ2/Kir2.1 regulates MDR, we first established two stable KCNJ2/Kir2.1-overexpressing sublines, H69-KCNJ2 and H446-KCNJ2, and suppressed KCNJ2/Kir2.1 expression in H69AR and H446AR cells by shRNAs specifically targeting *KCNJ2*. Next, we examined the effect of KCNJ2/Kir2.1 upregulation and downregulation on the sensitivity of SCLC cells to chemotherapeutic drugs (ADM, CDDP and VP-16). H69AR and H446AR cells became much more sensitive to chemotherapeutic agents than the NC groups after significantly inhibiting KCNJ2/Kir2.1 expression, whereas KCNJ2/Kir2.1 upregulation led to the desensitization of H69 and H446 cells to these drugs. Our findings indicate that KCNJ2/Kir2.1 is closely correlated with chemoresistance and may represent a potential clinical strategy for interfering with chemoresistance in SCLC; however, more clinical data are needed to verify this proposal.

To further investigate the possible mechanism of KCNJ2/Kir2.1 in SCLC chemoresistance, we evaluated the effect of KCNJ2/Kir2.1 on apoptosis and cell cycle control by flow cytometry. Our results indicated that one reason for the resistant phenotype of MDR SCLC cells may be that KCNJ2/Kir2.1 induces cell cycle arrest at the G0/G1 phase and inhibits drug-induced apoptosis. Moreover, consistent with the results obtained in vitro, KCNJ2/Kir2.1 promoted tumor growth in a xenograft nude mouse model. These results suggest that KCNJ2/Kir2.1 may play an oncogenic role in SCLC.

In addition to the potential therapeutic impact of KCNJ2/Kir2.1, our studies shed light on the mechanisms by which KCNJ2/Kir2.1 mediates MDR in SCLC. Several studies have confirmed that MRP1/ABCC1 is highly expressed in H69AR cells [30,31], and may be closely related to chemoresistance in SCLC [22]. Enyeart et al. showed that K^+^ channels, including KCNJ2/Kir2.1, might function with MRP1/ABCC1 [32]. In this study, we first found that MRP1/ABCC1 expression was positively correlated with KCNJ2/Kir2.1in SCLC cells and tissues. To further confirm this relationship, we performed Co-IP and demonstrated that Kir2.1 can interact with MRP1/ABCC1 in H69AR cells. Our data suggest that KCNJ2/Kir2.1 might affect the resistance to chemotherapy via interaction with MRP1/ABCC1 in SCLC cells.

The KCNJ2/Kir2.1 channel is modulated by several factors, including PKC, direct tyrosine kinase phosphorylation, the acidic intracellular pH and AMP-activated protein kinase [17,33-35]. The work reported by Giovannardi et al. has shown that RAS-PKC-MEK signaling is also an important regulator of KCNJ2/Kir2.1 [5]. In this study, KCNJ2/Kir2.1 expression at the mRNA and protein levels was markedly downregulated in the H69AR and H446AR cells after treatment with staurosporine, a PKC inhibitor, or U0126, a MEK inhibitor. It has been suggested that the KCNJ2/Kir2.1 channel is regulated by the Ras-MAPK pathway. However, whether Ras-MAPK signaling is involved in the mechanism by which KCNJ2/Kir2.1regulates SCLC MDR remains unknown.

miRNAs play an important role in the development of drug resistance in some tumor types [24,36], and our previous study showed that some miRNAs are involved in the development of drug resistance in SCLC cells [22]. Thus, we hypothesized that certain miRNAs could affect chemosensitivity by directly targeting KCNJ2/Kir2.1 in SCLC, and we identified miR-7 as a direct suppressor of KCNJ2/Kir2.1. Recently, miR-7 was reported to be a tumor suppressor due to its abilities to suppress cell growth and metastasis [37-39], promote apoptosis and inhibit drug resistance [40]. Our data showed that an miR-7 agomir or an antagomir led to a significant decrease or increase, respectively, in KCNJ2/Kir2.1 expression at both the mRNA and protein levels in SCLC cells. Luciferase reporter assays demonstrated that miR-7 directly targeted KCNJ2 in H69AR cells, and miR-7 expression was associated with SCLC chemoresistance. In addition, we found that miR-7 downregulation was associated with KCNJ2/Kir2.1 expression and advanced clinicopathological features of SCLC tissues. These findings indicate that KCNJ2/Kir2.1 is directly regulated by miR-7 in SCLC.

In summary, our findings reported here provide a novel mechanism by which KCNJ2/Kir2.1 modulates the sensitivity of SCLC cells to chemotherapeutic drugs, possibly through its regulation of MRP1/ABCC1 and simultaneous regulation by the Ras/MAPK pathway and miR-7. Therefore, our study indicates that KCNJ2/Kir2.1 may be a potential novel target for interfering with chemoresistance in SCLC.

## Methods and materials

### Tissue specimens

Fifty-two SCLC patient tissue samples were obtained from Zhujiang (Southern Medical University, Guangzhou, China) and Wujing Hospitals (Guangzhou Medical University, Guangzhou, China). All samples were confirmed as SCLC by pathologic examination and were further distinguished as limited disease (25 cases) or extensive disease (27 cases) according to the Veterans Administration Lung Study Group. All patients gave informed consent prior to the collection of specimens according to the institutional guidelines. Tissue samples were snap-frozen in the operating room immediately after surgery, and non-tumor tissues were sent to the pathology department for diagnosis by a board-certified pathologist. The non-tumor tissues (pericarcinomatous tissues) were confirmed to have surrounded the tumor tissue and to be free of cancer cells. A paraffin-embedded tissue specimen was available for each included patient. Under the protocol approved by the Institutional Review Board, informed consent was obtained from the patients or their guardians.

### Cells culture and transfection

Human SCLC H69 and H446 cell lines and the drug-resistant H69ARsubline were purchased from American Type Culture Collection (ATCC, USA). The other drug-resistant subline, H446AR, was established in our laboratory by culturing H446 cells in adriamycin (ADM). These cell lines were maintained in RPMI 1640 (GIBCO, Mississauga, Canada) supplemented with 10% heat-inactivated calf serum (HyClone, Logan, UT) and L-glutamine (Beyotime, Jiangsu, China) in an incubator at 37°C with 5% CO_2_. The H69AR and H446AR cell lines were challenged monthly for maintained resistance to the selected drugs, and their growth and morphology were monitored. The drug-resistant cells were maintained in drug-free medium for at least 2 weeks before any experiment.

For transient miRNAtransfection, cells were placed in standard media without antibiotics for 24 h before being transfected with anmiR-7agomir or antagomir or the corresponding negative controls (GenePharma, Shanghai, China) using Lipofectamine 2000 and OPTI-MEMI (Invitrogen) according to the manufacturer’s protocol. For stable transfections, the KCNJ2 coding region was inserted into pcDNA3.1 (GenePharma) andtransfected into H69 and H446 cells to stably overexpress KCNJ2 (H69-KCNJ2 and H446-KCNJ2). Cells stably transfected with the pcDNA3.1 empty expression vector (Invitrogen) were used as their corresponding negative controls (H69-NC and H446-NC). Positive transfectants were selected with 800 μg/ml geneticin (G418; Invitrogen). The *KCNJ2* gene was knocked down using two different KCNJ2 short-hairpin RNAs (shKCNJ2-1, shKCNJ2-2), which were obtained from GenePharma and transfected into H69AR and H446AR cells (H69AR-shKCNJ2-1, H69AR-shKCNJ2-2, H446AR-shKCNJ2-1 and H446AR-shKCNJ2-2) using Lipofectamine 2000. A negative control shRNA (shNC) was transfected into H69AR and H446AR cells (H69AR-shNC and H446AR-shNC) as the corresponding negative controls for cells transfected with shKCNJ2-1 or shKCNJ2-2. After the cells were treated for 24 h, G418 was used for 1 month to select the transfected cells. The short-hairpin RNAsequences are shown in Additional file 7: Table S2.

### Reagents and antibodies

A rabbit anti-human Kir2.1 polyclonal antibody was purchased from Alomone labs (Jerusalem, Israel), andpolyclonal anti-MRP1/ABCC1 and glyceraldehyde-3-phosphate dehydrogenase (GAPDH) monoclonal antibodies were purchased from Santa Cruz Inc. (CA, USA). Horseradish peroxidase (HRP)-labeled goat anti-rabbit immunoglobulin G (IgG) and goat anti-mouse IgG were obtained from Santa Cruz Inc. U0126, the MEK inhibitor, and staurosporine, the PKC inhibitor, were purchased from Selleck Chemicals (Houston, TX, USA). All three chemotherapeutic drugs, Cisplatin (DDP; Shandong, China), Etoposide (VP-16; Jiangsu, China) and Adriamycin (ADM; Jiangsu, China), were obtained from commercial sources and were dissolved according to the manufacturer’s instructions.

### RNA isolation, reverse transcription, and quantitative real-time PCR

Total RNA, including miRNA, was extracted from cell lines using TRIzol (Invitrogen) or the miRNeasy kit (Qiagen), according to the manufacturer’s instructions. For formalin-fixed, paraffin-embedded (FFPE) samples, total RNA was extracted from ten to fifteen 10-μm-thick sections usingmiRNeasy FFPE Kit (Qiagen). Total RNA was reverse transcribed using the PrimeScript RT reagent Kit (Takala, Dalian, China), andmiRNA sequence-specific reverse transcription (RT)-PCR for miR-7 and U6 was performed according to the Hairpin-itTMmiRNAs q-PCR quantitation kit and the U6 snRNA real-time PCR normalization kit (GenePharma). Quantitative real-time PCR was carried out using the MX3005 sequence detection system (Stratagene) with SYBR Green according to the manufacturer’s instructions. All primers are listed in Additional file 7: Table S3. GAPDH and U6 were used as endogenous controls. All samples were normalized to the internal controls, and fold changes were calculated through relative quantification (2^-△△Ct^) [41].

### Western blotting assay

For western blotting assays, total proteins were extracted from cells using RIPA lysis buffer (Sigma-Aldrich) and quantified using a BCA protein assay kit (Thermo). Total proteins were separated on 8% SDS–PAGE gels before being transferred to polyvinylidenedifluoride membranes (Bio-Rad). After the membranes were blocked with 5% non-fat milk, they were incubated with a rabbit anti-human Kir2.1 polyclonal or mouse anti-human MRP1/ABCC1 polyclonal antibody at 4°C overnight. GAPDH was used as a protein-loading control. After washing with Tris-buffered saline solution containing 0.1% Tween 20 (TBST, Bio-Rad), a peroxidase-linked secondary goat anti-mouse IgG or goat anti-rabbit IgG antibody was incubated with the membranes for 1 h at room temperature. After washing again with TBST, the protein bands were detected by chemiluminescence. The intensities of the protein bands were quantified with the Quantity One software (4.5.0 basic, Bio-Rad).

### Immunofluorescence staining

Cells were seeded into 24-well plates for 24 h before being fixed with paraformaldehyde at 4°C for 30 min. After being rinsed in PBS, the cells were incubated with 10% normal calf serum for 30 min to block non-specific IgG binding sites and then incubated with rabbit anti-human Kir2.1 monoclonal antibody (Alomone) (1:100 dilution) at 4°C overnight. A fluorescein isothiocyanate (FITC)-conjugated goat anti-rabbit IgGsecondary antibody (1:100 dilution) was added, and the cells were incubated for 1.5 h in the dark at room temperature. All images were captured using a fluorescence microscope (model Eclipse 660, Nikon, Japan).

### In vitro drug sensitivity assay

Cells were reseeded in 96-well plates at a density of 5 × 10^3^ per well and treated in medium with ADM, DDP or VP-16 for 24 h. Cell survival was then analyzed via the Cell Counting Kit-8 assay (CCK8, Dojindo Molecular Technologies, Japan) according to the manufacturer’s instructions. The range of drug concentrations was based on earlier studies [42] and was aimed to obtain IC_50_ values for both highly sensitive and resistant cases. After incubation with 10 μl of CCK-8 reagent for 4 h,the absorbance was measuredat 450 nm. The cells incubated without drugs were set at 100% survival and used to calculate the IC_50_ of each chemotherapeutic drug_._ The assay was carried out in six replicate wells for each sample, and three parallel experiments were conducted.

### Flow cytometric analysis

Cells were treated with drugs for 24 h and then collected for apoptosis and cell cycle analyses. Cell apoptosis assays were performed using an Annexin V/propidium iodide (PI) detection kit (Keygene, Nanjing, China) according to the manufacturer’s instructions. For cell cycle analysis, cells were harvested and fixed in 70% ethanol overnight at 4°C. After being washed three times in cold PBS, the cells were incubated with RNase and stained with PI. Cellquest Pro software was used for apoptosis analysis and ModFit LT software was used for analysis of cell cycle. Cells in the quadrant of Annexin V^−^PI^−^ (lower left) represent viable cells, cells in the quadrant of Annexin V^−^PI^+^ (upper left) mean necrotic cells, cells in the quadrant of Annexin V^+^PI^+^ (upper right) mean late apoptotic and dead cells and cells in the quadrant of Annexin V^+^PI^−^ (lower right) represent early apoptotic cells. Usually apoptosis analysis is mainly based on the percentages of Annexin V^+^PI^−^ cells. All assays were carried out independently in triplicate.

### Immunohistochemical analysis

Paraffin-embedded SCLC samples were sectioned and mounted on microscopic slides. Polyclonal anti-Kir2.1 and anti-MRP1/ABCC1 antibodies (Alomone Labs Ltd., Israel) was used as the primary antibodies. Antigen retrieval was performed by microwaving in 10 mmol/L citric acid buffer at pH 7.2. The samples were incubated first with the primary antibodies overnight at 4°C, then with the secondary antibodies for 2 h at room temperature in the same buffer and finally with abiotinylated secondary antibody (DAKO, Tokyo, Japan). The bound antibodies were visualized using the avidinbiotinylated peroxidase complex and diaminobenzidine tetrachloride methods (Santa Cruz Biotechnology).

The IHC-stained samples were independently evaluated for Kir2.1 and ABCC1 expressions by two pathologists blinded to the clinical parameters. The staining intensity was scored as 0 (negative), 1 (weak), 2 (medium) or 3 (strong). The extent of staining was scored as 0 (0-5%), 1 (6-25%), 2 (26-50%) or 3 (51-100%), according to the percentages of the positive stained area in relation to the entire carcinoma-involved area or the entire normal sample area. The sum of the intensity and extent scores was used as the final staining score (0–6). Optimal cut off values were identified; a final staining score ≤ 1 indicated negative expression and a final staining score >1 indicated positive expression.

### Luciferase reporter assay

The wildtype and mutated KCNJ23′UTR segments that were predicted to interact with miR-7 were amplified from human genomic DNA by PCR and inserted into psiCHECK-2 immediately downstream of the luciferase stop codon of (Promega) to develop psiCHECK2-KCNJ2-3′UTR and psiCHECK2-KCNJ2-mut-3′UTR. Cells in 24-well plates were transfected with psiCHECK2-KCNJ2-3′UTR, psiCHECK2-KCNJ2-mut-3′UTR or psiCHECK-2. Moreover, anmiR-7 agomir or antagomiror their corresponding negative control (NC agomir or NC antagomir) was also co-transfected into the cells. Luciferase activity was then assayed 48 h posttransfection using a dual-luciferase reporter assay system (Promega).

### Co-immunoprecipitation

Cells cultured in 10-cm dishes were harvested and lysed in 500 μl lysis buffer (20 mMTris (pH 7.4), 50 mMNaCl, 1 mM EDTA, 0.5% NP-40, 0.5% SDS, 0.5% deoxycholate, and protease inhibitors). Then, 500 μg lysate (1 μg/μl) was precleared with 50 μl protein A-Sepharose beads (Upstate Biotechnology, NY, USA) for 2 h at 4°C. An appropriate amount of rabbit anti-Kir2.1 antibody (Alomone) or rabbit non-specific IgG (Santa) was then added and incubated overnight at 4°C. Then, 100 μl preblocked agarose beads was added to the antibody/lysate mixture and incubated for another 2 h at 4°C; the beads were then pelleted and washed twice with lysis buffer. Both the cell lysates without IP and the immunoprecipitates were eluted in SDS sample buffer, subjected to SDS-PAGE and analyzed by immunoblotting.

### In vivo tumor xenograft model

Six- to eight-week-old female BALB/c nude mice (purchased from the Medical Experimental Animal Center of Guangdong Province, China) were used for *in vivo* assays. The mice were raised under pathogen-free conditions, and all procedures were performed according to the guidelines of the Association for the Assessment and Accreditation of Laboratory Animal Care International. Cells were harvested, washed with PBS and re-suspended in normal culture medium at a concentration of 1 × 10^7^ cells/0.1 ml. Cells in RPMI 1640 were subcutaneously inoculated into the legs of nude mice to establish the tumor model. The tumor volume was determined three times per week by direct measurement with a sliding caliper and was calculated using the following equation: V = (4/3) × π × R1^2^× R2, where R1 is radius 1, R2 is radius 2, and R1 < R2. Growth curves of the tumors were constructed. After 20 days, 5 mice from each group were sacrificed, and the tumors were excised and fixed with neutral phosphate-buffered formalin. Subsequently, consecutive tissue sections from the tumors were sliced and then stained with hematoxylin-eosin.

### Statistical analysis

All experiments were run in triplicate, and the results are presented as the mean ± SD. Statistical analyses were performed using either an analysis of variance (ANOVA) or Student's *t* test. The association between Kir2.1or MRP1/ABCC1 or miR-7expression and clinical features was analyzed by *χ*^2^ test. The relationship between Kir2.1 and MRP1/ABCC1 was explored by *χ*^2^ test. Survival curves were obtained by Kaplain-Meier analysis. The positive rate of Kir2.1 and MRP1/ABCC1 in normal lung tissue was compared with that in SCLC tissue by *χ*^2^ test. A difference was considered statistically significant when the P value was less than 0.05. All statistical analyses were carried out with SPSS 17.0 software.

## Additional files

Additional file 1: Figure S1. The semiquantitative analyses of IHC for Kir2.1 and ABCC1 expression. **(A)** The final staining scores of IHC in Figure 1A-D for Kir2.1 and ABCC1 expression in SCLC and normal lung tissues. **(B)** The final staining scores of IHC in Figure 6E for Kir2.1 expression. Additional file 2: Figure S2. Altered expression of KCNJ2/Kir2.1 affects the cell cycle distribution of SCLC cells after treatment with CDDP. **(A)** Representative FACS profiles are shown, on which numbers indicate percentage of cells in G0/G1, S or G2/M phase. **(B)** The histographs for cell cycle distribution of SCLC cells. Data are shown as means ± SD from three independent experiments. *, *P* < 0.05; **, *P* < 0.01 compared to the corresponding NC cells transfected with pcDNA3.1 empty vector or shNC. Additional file 3: Figure S3. Altered expression of KCNJ2/Kir2.1 affects the cell cycle distribution of SCLC cells after treatment with VP-16. **(A)** Representative FACS profiles are shown, on which numbers indicate percentage of cells in G0/G1, S or G2/M phase. **(B)** The histographs for cell cycle distribution of SCLC cells. Data are shown as means ± SD from three independent experiments. *, *P* < 0.05; **, *P* < 0.01 compared to the corresponding NC cells transfected with pcDNA3.1 empty vector or shNC. Additional file 4: Figure S4. Altered expression of KCNJ2/Kir2.1 affects cell apoptosis in SCLC cells after treatment with CDDP. **(A)** Representative FACS profiles are shown. **(B)** The histographs for cell apoptosis of SCLC cells. The results show data from at least three independent experiments. *, *P* < 0.05; **, *P* < 0.01 compared to the corresponding NC cells. Additional file 5: Figure S5. Altered expression of KCNJ2/Kir2.1 affects cell apoptosis in SCLC cells after treatment with VP-16. **(A)** Representative FACS profiles are shown. **(B)** The histographs for cell apoptosis of SCLC cells. The results show data from at least three independent experiments. *, *P* < 0.05; **, *P* < 0.01 compared to the corresponding NC cells. Additional file 6: Figure S6. Kaplan–Meier analysis of overall survival of 52 patients with SCLC based on miR-7 expression. (*P* < 0.05). Additional file 7: Table S1. Univariate analysis of overall survival with regard to clinicopathological characteristics. **Table S2.** The sequences of short hairpin RNA used in vector construction. **Table S3.** Primers used in real-time quantitative RT-PCR.

## Abbreviations

IHC: Immunohistochemistry
MRP1/ABCC1: Multidrug resistance protein 1
MDR: Multidrug resistance
MiRNAs: microRNAs
GAPDH: Glyceraldehyde-3-phosphate dehydrogenase
