# Supplementary figures and images for: Upregulation of the inwardly rectifying potassium channel Kir2.1 (KCNJ2) modulates multidrug resistance of small-cell lung cancer under the regulation of miR-7 and the Ras/MAPK pathway
Source: Mol Cancer. 2015 Mar 12;14:59. doi: 10.1186/s12943-015-0298-0 (PMC4373128; doi:10.1186/s12943-015-0298-0)

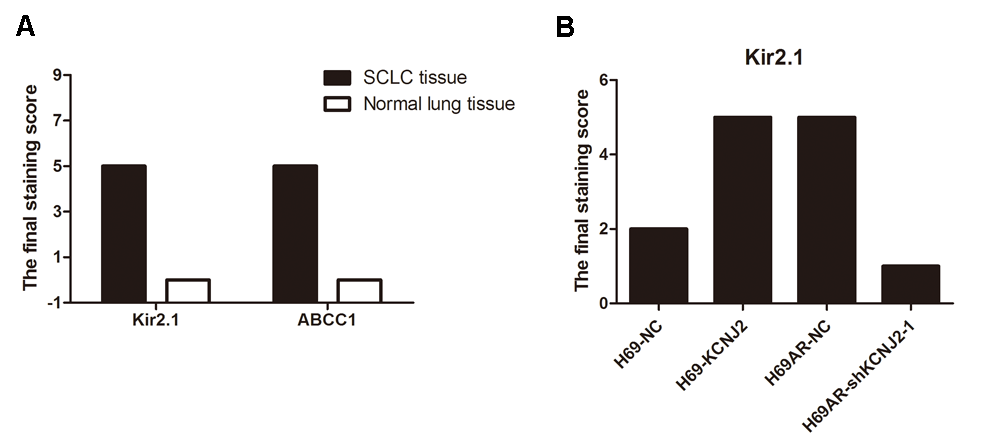

Supplement: Additional file 1: Figure S1. — The semiquantitative analyses of IHC for Kir2.1 and ABCC1 expression. (A) The final staining scores of IHC in Figure 1A-D for Kir2.1 and ABCC1 expression in SCLC and normal lung tissues. (B) The final staining scores of IHC in Figure 6E for Kir2.1 expression. [file 12943_2015_298_MOESM1_ESM.tiff]

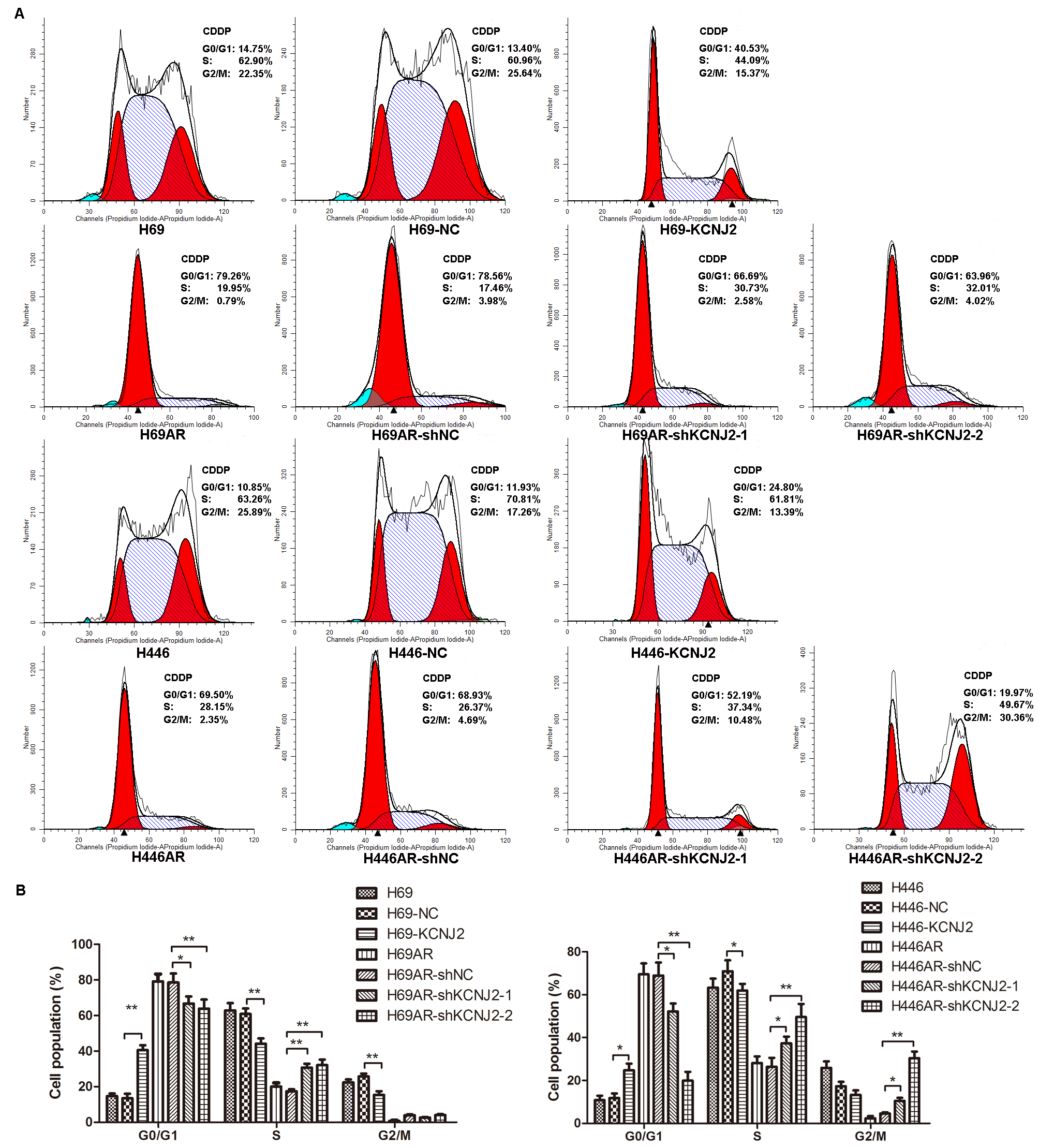

Supplement: Additional file 2: Figure S2. — Altered expression of KCNJ2/Kir2.1 affects the cell cycle distribution of SCLC cells after treatment with CDDP. (A) Representative FACS profiles are shown, on which numbers indicate percentage of cells in G0/G1, S or G2/M phase. (B) The histographs for cell cycle distribution of SCLC cells. Data are shown as means ± SD from three independent experiments. *, P < 0.05; **, P < 0.01 compared to the corresponding NC cells transfected with pcDNA3.1 empty vector or shNC. [file 12943_2015_298_MOESM2_ESM.tiff]

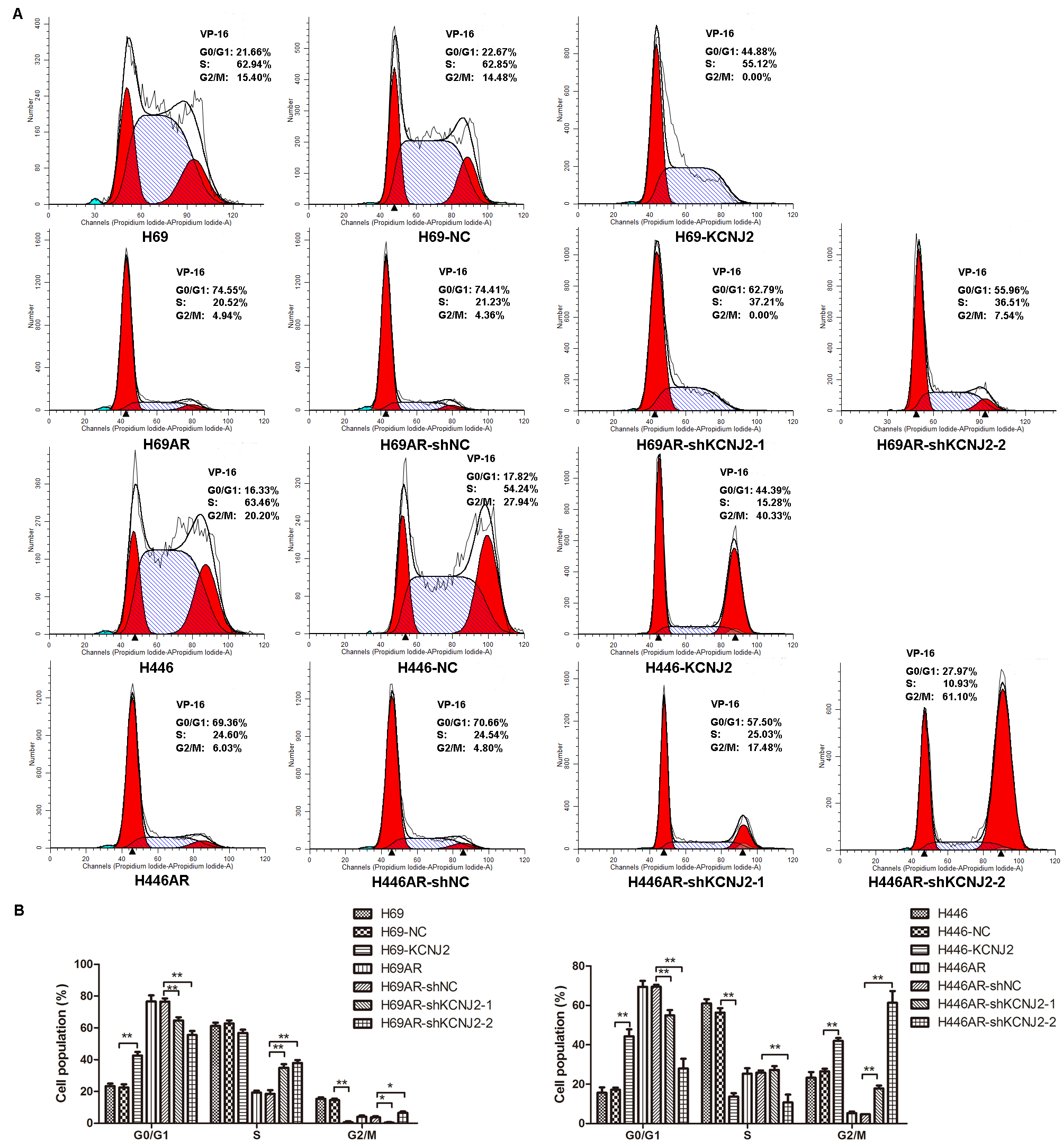

Supplement: Additional file 3: Figure S3. — Altered expression of KCNJ2/Kir2.1 affects the cell cycle distribution of SCLC cells after treatment with VP-16. (A) Representative FACS profiles are shown, on which numbers indicate percentage of cells in G0/G1, S or G2/M phase. (B) The histographs for cell cycle distribution of SCLC cells. Data are shown as means ± SD from three independent experiments. *, P < 0.05; **, P < 0.01 compared to the corresponding NC cells transfected with pcDNA3.1 empty vector or shNC. [file 12943_2015_298_MOESM3_ESM.tiff]

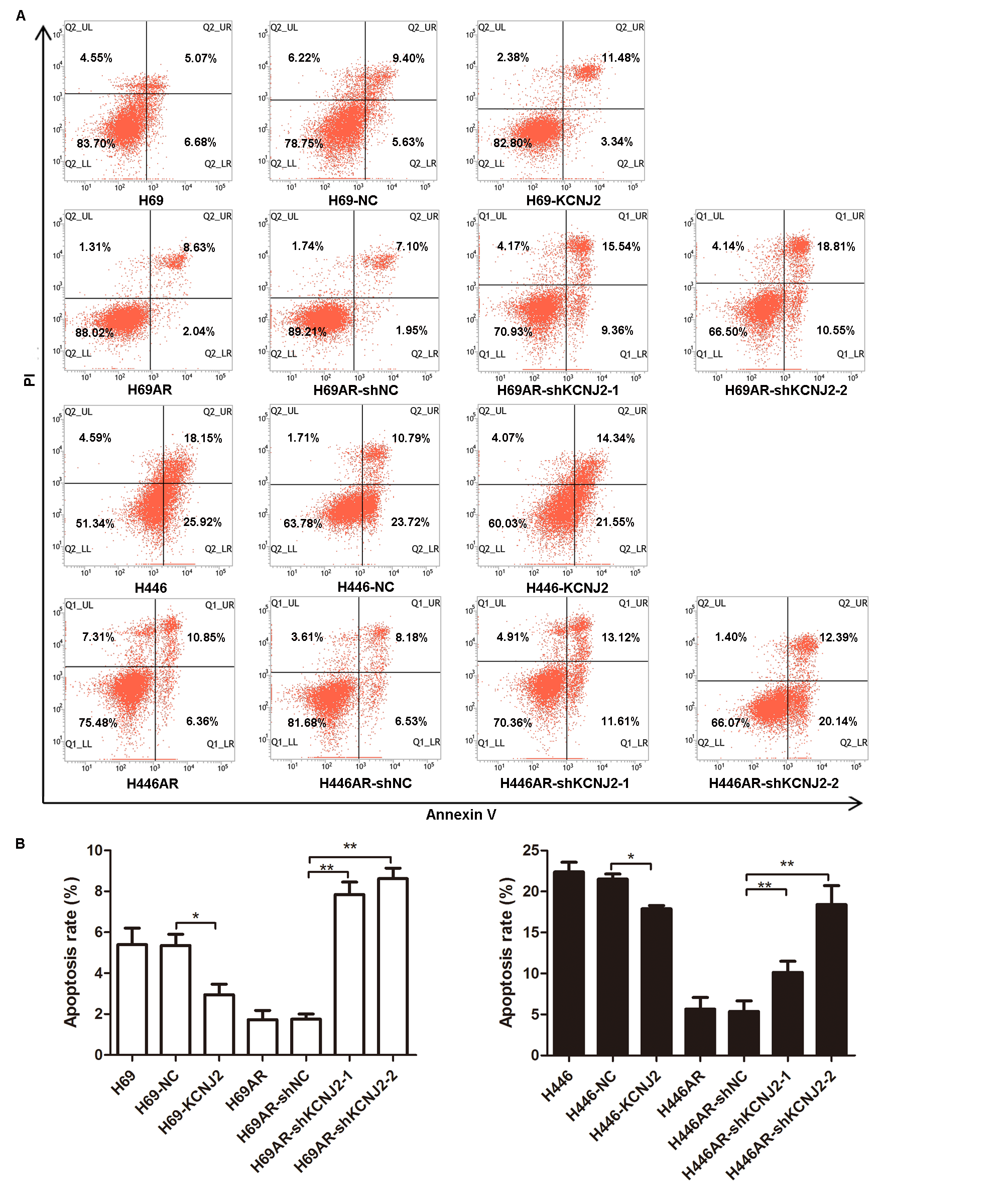

Supplement: Additional file 4: Figure S4. — Altered expression of KCNJ2/Kir2.1 affects cell apoptosis in SCLC cells after treatment with CDDP. (A) Representative FACS profiles are shown. (B) The histographs for cell apoptosis of SCLC cells. The results show data from at least three independent experiments. *, P < 0.05; **, P < 0.01 compared to the corresponding NC cells. [file 12943_2015_298_MOESM4_ESM.tiff]

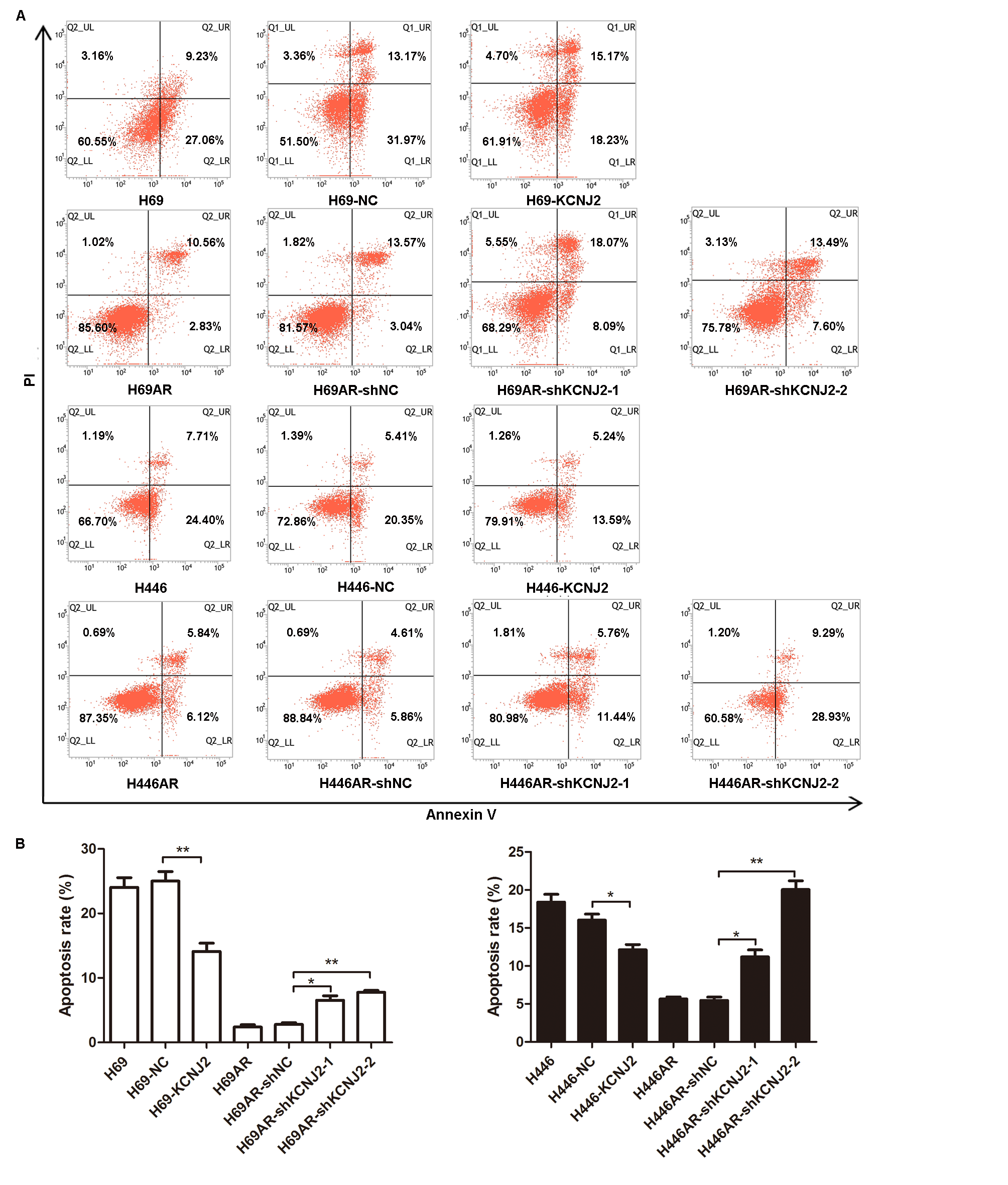

Supplement: Additional file 5: Figure S5. — Altered expression of KCNJ2/Kir2.1 affects cell apoptosis in SCLC cells after treatment with VP-16. (A) Representative FACS profiles are shown. (B) The histographs for cell apoptosis of SCLC cells. The results show data from at least three independent experiments. *, P < 0.05; **, P < 0.01 compared to the corresponding NC cells. [file 12943_2015_298_MOESM5_ESM.tiff]

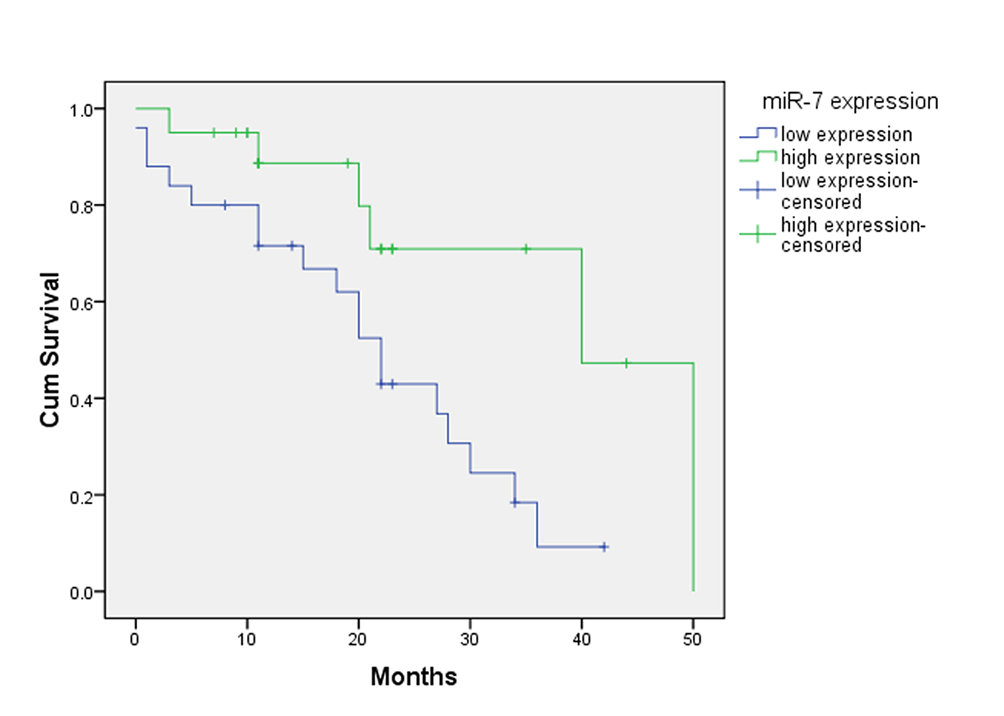

Supplement: Additional file 6: Figure S6. — Kaplan–Meier analysis of overall survival of 52 patients with SCLC based on miR-7 expression. (P < 0.05). [file 12943_2015_298_MOESM6_ESM.tiff]
