# Supplementary material for: Upregulation of the inwardly rectifying potassium channel Kir2.1 (KCNJ2) modulates multidrug resistance of small-cell lung cancer under the regulation of miR-7 and the Ras/MAPK pathway
Source: Mol Cancer. 2015 Mar 12;14:59. doi: 10.1186/s12943-015-0298-0 (PMC4373128; doi:10.1186/s12943-015-0298-0)
Supplement: Additional file 7: Table S1. — Univariate analysis of overall survival with regard to clinicopathological characteristics. Table S2. The sequences of short hairpin RNA used in vector construction. Table S3. Primers used in real-time quantitative RT-PCR. [file 12943_2015_298_MOESM7_ESM.docx]

**Supplementary Tables**

**Table S1. Univariate analysis of overall survival with regard to clinicopathological characteristics**

| Characteristics | Univariate Analysis | |
| --- | --- | --- |
|  | *HR*(95% CI) | *P* |
| Gender | 0.657(0.193-2.236) | 0.502 |
| Age | 0.337(0.142-0.802) | **0.014** |
| Stage | 1.664(0.701-3.949) | 0.248 |
| Drug-sensitivity | 1.810(0.787-4.159) | 0.162 |
| MiR-7 | 0.315 (0.115-0.858) | **0.024** |
| KCNJ2 | 1.516 (0.663-3.468) | 0.324 |
| ABCC1 | 1.743 (0.738-4.114) | 0.205 |

Significant differences are shown in bold.

**Table S2. The sequences of short hairpin RNA used in vector construction**

| **Name** |  | **ShRNA sequence** |
| --- | --- | --- |
| **shNC** | Sense | 5’-CACCGTTCTCCGAACGTGTCACGTCAAGAGATTACGTGACACGTTCGGAGAATTTTTTG-3’ |
|  | Antisense | 5’-GATCCAAAAAATTCTCCGAACGTGTCACGTAATCTCTTGACGTGACACGTTCGGAGAAC-3’ |
| **ShKCNJ2-1** | Sense | 5’-CACCGGTGGATGCTGGTTATCTTCTTTCAAGAGAAGAAGATAACCAGCATCCACCTTTTTTG-3’ |
|  | Antisense | 5’-GATCCAAAAAAGGTGGATGCTGGTTATCTTCTTCTCTTGAAAGAAGATAACCAGCATCCACC-3’ |
| **ShKCNJ2-2** | Sense | 5’-CACCGCTCCTCAAATCCAGAATTACTTCAAGAGAGTAATTCTGGATTTGAGGAGCTTTTTTG-3’’ |
|  | Antisense | 5’-GATCCAAAAAAGCTCCTCAAATCCAGAATTACTCTCTTGAAGTAATTCTGGATTTGAGGAGC-3’ |

**Table S3. Primers used in real-time quantitative RT-PCR**

| **Gene** |  | **Primer sequences** |
| --- | --- | --- |
| **KCNJ2** | Forward | 5’-TGGATGCTGGTTATCTTCTGC-3’ |
|  | Reverse | 5’-AGCCTATGGTTGTCTGGGTCT-3’ |
| **ABCC1** | Forward | 5’-GTCATCCTTGCTCTCTACCTCCT-3’ |
|  | Reverse | 5’-CCTGATACGTCTTGGTCTTCATC-3’ |
| **GAPDH** | Forward | 5’-AGAAGGCTGGGGCTCATTTG-3’ |
|  | Reverse | 5’-AGGGGCCATCCACAGTCTTC-3’ |
